# High-purity 1,2-dimyristoyl-*sn*-glycero-3-phosphocholine: synthesis and emulsifying performance evaluation

**DOI:** 10.3389/fnut.2024.1408937

**Published:** 2024-07-09

**Authors:** Se-Young Kim, Ye-Lim Park, Ha-Eun Ji, Hae-Se Lee, Hyeon-Jun Chang, Gyeong-Hee Bang, Jeung-Hee Lee

**Affiliations:** ^1^Department of Food Science and Technology, Chungnam National University, Daejeon, Republic of Korea; ^2^Department of Food and Nutrition, Daegu University, Gyeongsan-Si, Republic of Korea

**Keywords:** 1,2-dimyristoyl-*sn*-glycero-3-phosphocholine, purification, emulsion stability, emulsifier, simulated intestinal digestion, *in vitro* digestion, controlled release

## Abstract

**Introduction:**

1,2-Dimyristoyl-*sn*-glycero-3-phosphocholine (DMPC) is a promising emulsifier for bioactive delivery systems, but its industrial applications are limited by the lack of cost-effective and scalable synthetic routes. The purpose of this study was to economically produce high-purity DMPC by replacing commonly used column chromatography methods and to evaluate the emulsifying performance.

**Methods:**

DMPC was synthesized from *sn*-glycero-3-phosphocholine using Steglich esterification followed by sequential recrystallization from ethyl acetate and acetone. The structure of DMPC was identified and its purity was confirmed using various spectroscopy and chromatography techniques. The emulsifying performance was evaluated by examining the effects of storage on the properties of o/w emulsions prepared using soybean oil with (i) soy PC, (ii) soy PC + DMPC (1:1, w/w), and (iii) DMPC as emulsifiers.

**Results:**

The chemical impurities formed during the synthesis of DMPC was removed, and its final purity was 96%, and the melt transition temperature was 37.6°C. No visible difference between the three emulsions (soy PC, soy PC+DMPC, and DMPC) was observed during two-week storage, and the DMPC-based emulsion was more stable than soy PC emulsion, showing smaller particle size distribution during 6 months.

**Discussion:**

The highly pure DMPC was synthesized by an economical method, and DMPC-based emulsions demonstrated physicochemical stable, highlighting its potential for food and pharmaceutical industry-related applications. Our findings suggest that DMPC holds promise as an emulsifier with broad applications in the food industry.

## 1 Introduction

Emulsions are colloidal systems that are widely used for oral and parenteral delivery applications in the food and pharmaceutical industries (1, 2) and can be divided into water-in-oil (w/o), oil-in-water (o/w), microemulsions and multiple emulsions. Generally, the oil droplets in o/w emulsions consist of an oil core surrounded by emulsifier molecules and can be loaded with substantial amounts of lipophilic bioactive substances. Moreover, emulsions are well suited for large-scale industrial production (3). However, the widespread use of o/w emulsions as delivery systems is hindered by their low physicochemical stability. The encapsulation of lipophilic substances intended for oral administration in oil droplets provides protection against the harsh environment of the gastrointestinal tract (e.g., pH and digestive enzymes) and enables the efficient introduction of bioactive agents into the body through targeted release based on digestibility modulation (2, 4, 5). The physical stability of oil droplets and their susceptibility to enzymatic degradation in the gastrointestinal tract are determined by their interfacial properties.

On the other hand, emulsions are thermodynamically unstable because free energy increases due to the contact between the oil phase and the water phase compared to when the phases exist individually. Such thermodynamic instability of emulsions arises from a decrease in entropy of mixing, which leads to an increase in free energy (6). This instability leads to phase separation over time through flocculation, coalescence, and Ostwald ripening. Even an o/w emulsion can undergo phase inversion to become a w/o emulsion, typically triggered by changes in composition or temperature. Therefore, stable emulsions can be obtained by using an emulsifier that effectively reduces interfacial tension by adsorption of monolayer on the oil–water interface. Emulsifiers used in food industry can be largely divided into two categories: low molecular weight emulsifiers such as phospholipids and polysorbates, and amphiphilic biopolymers (e.g., whey protein, caseins) (7, 8).

Phosphatidylcholines (PCs) are widely used low-molecular-weight emulsifiers comprising a hydrophilic head group (choline) and two hydrophobic tails (fatty acid residues) (9). Soybean lecithin, the main emulsifier used in the food industry, is a low-purity PC as a mixture of various phospholipids. Higher-purity PCs (e.g., those for the pharmaceutical industry) can be isolated from egg yolk, as it is richer in PCs than soybeans, which results in easier separation. The glycerol backbone of natural PCs is predominantly ester-bonded to two different fatty acids, e.g., linoleic acid (C18:2n-6) and oleic acid (C18:1n-9) (10–12). Given the unsaturated nature of these fatty acids, the temperature of the main phase transition (*T*_m_), i.e., the conversion of the gel phase (L*_β_*) into the liquid-crystalline phase (L*_α_*), is less than 0°C (12). In addition, the rigid *cis* double bonds in the unsaturated fatty acid residues lower the packing density of the acyl chains and therefore increase interfacial fluidity and favor the formation of lipid packing defects (13). As a result, only L*_α_* appears at ≥0°C, which complicates the realization of release kinetics tailored to the needs of particular applications.

Several formulations (e.g., vaccines and drugs for intramuscular, subcutaneous, and intravenous administration) with various release kinetics have been developed based on synthetic PCs, which have higher *T*_m_ values than natural PCs (14–16). The *T*_m_ of a given PC depends on its fatty acid residues and influences fluidity and permeability, thus playing an important role in the regulation of release kinetics. Among the synthetic PCs, saturated ones, such as 1,2-dimyristoyl-*sn*-glycero-3-phosphocholine (DMPC), 1,2-dipalmitoyl-*sn*-glycero-3-phosphocholine (DPPC), and 1,2-distearoyl-*sn*-glycero-3-phosphocholine (DSPC), have drawn particular attention. Depending on temperature, saturated PCs generally exist in four lamellar phases, namely the liquid-crystalline phase (L*_α_*), ripple gel phase (P*_β_*), gel phase (L*_β_*), and subgel or crystalline phase (L*_c_*). The *T*_m_ (temperature of the P*_β_* ↔ L*_α_* interconversion) of DMPC, DPPC, and DSPC is 23, 41.5, and 54.5°C, respectively. Therefore, at body temperature, DMPC, DPPC, and DSPC are present as L*_α_*, P*_β_*, and L*_β_* and therefore exhibit different release profiles in the body (17–19).

DMPC exists in a gel phase at 4°C and a liquid-crystalline phase at body temperature. In a recent study on PC-stabilized emulsions, the mean droplet diameter was shown to increase upon going from DMPC (144.8 nm) to DSPC (344.5 nm) and decrease with the decreasing number of carbons in the fatty acid (20). This finding suggests that DMPC can be used to fabricate stable emulsions with controllable release behavior.

Common DMPC syntheses rely on tedious and time-consuming chromatographic purification, resulting in high costs for commercially available DMPC due to labor and expenses. While the small quantities required at the research stage make the high cost of synthetic phospholipids less problematic using column chromatography, the cost would become a significant barrier during the development stage when scale-up necessitates larger quantities. Hence, cheaper and faster alternatives should be developed to enable the industrial applications of DMPC. Moreover, the produced DMPC should be of sufficiently high purity, particularly if intended for use in the pharmaceutical industry. Herein, DMPC was synthesized through the Steglich esterification of silica-immobilized *sn*-glycero-3-phosphocholine (GPC) and myristic acid as reaction substrates. Subsequently, high-purity DMPC was produced through sequential crystallization for liquid–liquid extraction and purification, which is much more economical than conventional methods. The purified product was used to prepare DMPC-, PC from soybean [soy PC]-, and [DMPC + soy PC]-stabilized emulsions with soybean oil as the dispersed phase, and these emulsions were compared in terms of the fluidity and permeability of their o/w interface and hydrolyzability of their oil droplets under simulated small-intestinal digestion conditions.

## 2 Materials and methods

### 2.1 Materials

GPC and soy PC were procured from Solus Advanced Materials (Seoul, South Korea). Myristic acid (≥99%), pancreatin from porcine pancreas, lipase from porcine pancreas (Type II), bile salts, and bovine serum albumin were purchased from Sigma-Aldrich Co., Ltd. (St. Louis, MO, United States). Silica gel 60 (0.063–0.200 mm) was purchased from Merck KGaA (Darmstadt, Germany), 4-dimethylaminopyridine (DMAP) was purchased from Daejung Chemicals & Metals Co., Ltd. (Siheung, South Korea), and *N*, *N*′-dicyclohexylcarbodiimide (DCC) was purchased from Tokyo Chemical Industry Co., Ltd. (Tokyo, Japan). The DMPC standard was purchased from Avanti Polar Lipids, Inc. (Birmingham, AL, United States). Soybean oil was purchased from Lotte Foods (Cheonan, South Korea). *n*-Hexane, isopropanol, and water used for high-performance liquid chromatography (HPLC) were purchased from Fisher Scientific International, Inc. (Waltham, MA, United States), and acetonitrile, isopropanol, and ammonium acetate were purchased from Sigma-Aldrich (St. Louis, MO, United States).

### 2.2 Synthesis and purification of DMPC

DMPC was synthesized using a modification of a previously reported method (21). In order to increase the reaction yield, the reaction temperature was increased by 15°C and the reaction time was extended by more than fivefold compared to the previous method. A solution of GPC (2.65 g, 10.3 mmol) in methanol (8.8 mL) was dropwise added to silica gel (7.93 g) to prepare a silica-GPC complex, which was vacuum-concentrated at 40°C for 1 h and 80°C for 1 h. A screw flask was charged with a mixture of this complex, 100 mL of chloroform, 10.96 g of myristic acid, 9.90 g of DCC, and 3.05 g of DMAP (GPC: myristic acid: DCC: DMAP = 1.0: 4.8: 4.8: 2.5, mol/mol/mol/mol), purged with N_2_, sealed, and heated at 45°C for 72 h upon stirring at 550 rpm. The reaction mixture was vacuum-filtered through Whatman filter paper (grade 4) to partly remove dicyclohexylurea (DCU), and the chloroform filtrate was collected (Fraction 1). The filter cake was resuspended in chloroform (100 mL), and the dispersion was stirred for 1 h. The collected chloroform layer (Fraction 2) was mixed with Fraction 1, and the combined organic phase was treated with an equal amount of a 0.25 N HCl: methanol solution (1:1, v/v) for 3 min to remove DMAP and GPC. The process was repeated twice. The organic phase was supplemented with methanol (240 mL) and water (320 mL), and the mixture was agitated for 3 min. The resulting HCl-free chloroform layer was collected and vacuum-concentrated. The concentrate was mixed with an 18-fold (by weight) amount of ethyl acetate, and the mixture was stirred at 50°C for 1 h and then left to stand at 4°C for 30 min to precipitate DMPC. DCC, fatty acid anhydrides, myristic acid, and other byproducts were removed by centrifugation (3,000 rpm, 3 min), which was repeated four times. The resulting precipitate was dispersed in chloroform (50°C) to a concentration of 150 mg/mL. The dispersion was filtered through a hydrophobic syringe filter (0.50 μm), supplemented with acetone, and the mixture was maintained at −6°C while agitated for 1 h at 150 rpm. The precipitate was collected by centrifugation (2,500 rpm, 5 min), and the solvent was removed using N_2_ to obtain pure DMPC.

### 2.3 Nuclear magnetic resonance spectroscopy

For ^1^H and ^13^C NMR analysis, the sample (~10 mg) was dissolved in CDCl_3_ (0.7 mL), and the solution was passed through a Pasteur pipette filled with anhydrous sodium sulfate and placed in an NMR tube. NMR spectra (Bruker Avance III-600, Bruker BioSpin, Billerica, MA, United States) were recorded under the following conditions: acquisition time = 2.656 s, spectral width = 12335.5 Hz, number of scans = 16, frequency = 600 MHz (^1^H) or 150 MHz (^13^C). Chemical shifts (*δ*) were referenced to tetramethylsilane (*δ* = 0 ppm).

For ^31^P NMR analysis, the sample was supplemented with a solution of triphenyl phosphate in CDCl_3_ (1 mL, 1 mg/mL), methanol (0.5 mL), and EDTA-Na^+^ solution (0.5 mL, 200 mM, pH 7.6), and the mixture was vortexed for 2 min and then centrifuged (3,000 rpm, 10 min). The subnatant was collected, passed through an anhydrous sodium sulfate column, and placed in an NMR tube. NMR spectra were recorded using the instrument employed for ^1^H and ^13^C measurements. An inverse gating decoupling method was used to prevent the nuclear overhauser effect. ^31^P NMR spectra were acquired under the following conditions: probe temperature = 25°C, excitation pulse = 30°, number of datapoints = 64 K, relaxation delay = 2 s, pulse width = 11.05 μs, acquisition time = 0.34 s, number of scans = 256.

### 2.4 Fourier transform infrared spectroscopy

FTIR spectra were recorded in the 4,000–400 cm^−1^ range using a VORTEX 80v instrument (Bruker BioSpin, Billerica, MA, United States) with a deuterated triglycine sulfate detector.

### 2.5 HPLC analysis

DMPC was analyzed using an HPLC system equipped with a LiChrospher 100 DIOL column (5 μm, 250 mm × 4 mm, Merck, Darmstadt, Germany) and an evaporative light scattering detector (ELSD; ZAM-3000, Schambeck SFD, Bad Honnef, Germany). The oven temperature, drift tube temperature, pressure, and injection volume were set at 40°C, 60°C, 1.6 Standard Liter per Minute, and 20 μL, respectively. The solvent system was composed of solvents A (*n*-hexane: isopropanol: acetic acid: triethylamine = 81.42: 17.00: 1.50: 0.08, v/v/v/v) and B (isopropanol: water: acetic acid: triethylamine 84.42: 14.00: 1.50: 0.08, v/v/v/v). The linear gradient used for elution was as follows: 0–3.0 min, 0% B; 3.0–8.0 min, 0–5% B; 8.0–15.0 min, 5–20% B; 15.0–30.0 min, 20–30% B; 30.0–35.0 min, 30–40% B; 35.0–43.0 min, 40–80% B; 43.0–45.0 min, 80–100% B; 45.0–50.0 min, 100% B; 50.0–55.0 min, 100–0% B; 55.0–60.0 min, 100% B.

### 2.6 Ultra-performance liquid chromatography–tandem mass spectrometry analysis

A UPLC instrument (ACQUITY UPLC H-Class Core System, Waters, Milford, MS, United States) interfaced with a mass spectrometer (Xevo TQ-S micro, Waters, Milford, MS, United States) and an ACQUITY UPLC BEH C18 column (1.8 mm × 50 mm, 1.7 μm, Waters, Milford, MS, United States) was used. Mass spectra were recorded under the following conditions: source temperature = 150°C, flow rate = 0.2 mL/min, capillary voltage = 3.5 kV, cone voltage = 30 V, *m*/*z* range = 100–1700. The concentrations of the standard and final DMPC were 0.1 and 0.5 mg/mL, respectively. The solvent system was composed of solvents A (acetonitrile: 10 mM aqueous ammonium acetate (60:40, v/v)) and B (isopropanol: 10 mM acetonitrilic ammonium acetate (90:10, v/v)). The linear gradient elution program was as follows: 0–2.0 min, 15–30% B; 2.0–2.5 min, 30–48% B; 2.5–8.5 min, 48–72% B; 8.5–11.5 min, 72–99% B; 11.5–12 min, 99% B; 12–12.1 min, 99–15% B.

### 2.7 Matrix-assisted laser desorption and ionization time-of-flight mass spectrometry analysis

An Autoflex maX instrument (Bruker Daltonics, Bremen, Germany) equipped with a 200 Hz Nd YAG pulsed laser (*λ* = 355 nm) was used for MALDI-TOF-MS analysis. The extraction voltage equaled 20 kV, and a multiple-channel plate detector was used to acquire positive-ion spectra in the reflector mode. The sample was dissolved in the Folch solution (chloroform: methanol (2:1, v/v)), and the dispersion was supplemented with 2,5-dihydroxybenzoic acid in methanol (1:1, v/v) and loaded (1 μL) on the target plate.

### 2.8 Differential scanning calorimetry analysis

The phase transition characteristics of DMPCs (standard and synthesized) and soybean oil in water emulsion (5 wt%) with DMPC (0.5 wt% as emulsifier) were analyzed using DSC (Perkin Elmer, Waltham, MA, United States). The sample (1–5 mg for DMPC and 7–10 mg for emulsion) was placed in an aluminum pan. The sample was heated from 5°C to 50°C at 5°C/min, and a DSC melting thermogram was obtained.

### 2.9 Emulsion stability

Three o/w emulsions, i.e., those with soy PC (100%), DMPC (100%), and soy PC + DMPC (each 50%) as emulsifiers, were prepared. The emulsifier 0.25 g (0.5 wt%) was dissolved in distilled water upon magnetic stirring at 75–80°C for 2 h, and the solution was supplemented with soybean oil 2.5 g (5 wt%) and ultrasonicated (GE 750, Sonics & Materials, Inc., Newtown, CT, United States) at a duty ratio of 95% for 1 min (65°C, four times). The prepared o/w emulsions were stored for 14 days (4 and 25°C) or 6 months (4°C). After 6 months of storage, the emulsion droplets were observed using optical microscopy (Olympus BX53, Tokyo, Japan). A polarizer (Olympus U-POT polarizer) was used to examine DMPC crystallization. Additionally, the phase volume ratio of each emulsion was observed. The emulsion droplet size was measured using a particle size analyzer (Malvern Master Sizer S, Malvern Co., Worcestershire, United Kingdom) and represented by the weighted average mean diameter (*D*[4, 3]) and particle size distribution. The zeta potentials of the emulsion droplets were measured at 37°C (Zetasizer Nano ZS, Malvern Instruments Ltd., Malvern, United Kingdom). The emulsion was diluted 100-fold with pH 5.8 deionized water and pH 7.8 buffer and placed in a folded capillary cell (Malvern Instruments, Malvern, United Kingdom). Since liver bile is generally known to have a pH 7.8 (22), bile salts were dissolved in a pH 7.8 buffer and added at a loading typically used for *in vitro* digestion (1.167 mg bile salt in 1 mg oil).

### 2.10 *In vitro* pH-stat digestion

A pH-stat *in vitro* digestion model was used to simulate the small-intestinal digestion of emulsions according to a modification of the method of Versantvoort et al. (23). The quantities of inorganic and organic solvents were maintained consistent with the previous method, while the amounts of the digestive enzymes (pancreatin and pancreatic lipase) were reduced to enable more detailed measurements of the differences in *in vitro* digestion. Pancreatin and pancreatic lipase were each used at concentrations of 3.5 wt% and 14 wt%, respectively, as per the previous method. An automatic potentiometric titrator (AT-400E, Kyoto Electronics Manufacturing Co., Ltd., Japan) and an auto piston burette (APB-410, Kyoto Electronics Manufacturing Co., Ltd., Japan) were used (4). Table 1 lists the compositions of the simulated duodenal and bile juices. The digestion fluid was prepared by mixing the duodenal (24 mL) and bile (12 mL) juices. The enzyme solution was prepared by dissolving pancreatin (108 mg) and lipase (72 mg) in the digestion fluid (1 mL), and 0.07 mL of this solution was added to a digestion cell (100 mL beaker) for hydrolysis.

**Table 1:** Composition of duodenal and bile juice for *in vitro* pH-stat digestion.

	Duodenal juice	Bile juice
Inorganic components	4 mL NaCl (175.3 g/L)	3 mL NaCl (175.3 g/L)
	4 mL NaHCO_3_ (84.7 g/L)	6.83 mL NaHCO_3_ (84.7 g/L)
	1 mL KH_2_PO_4_ (8 g/L)	0.42 mL KCl (89.6 g/L)
	0.63 mL KCl (89.6 g/L)	
	1 mL MgCl_2_ (5 g/L)	
Organic components	0.4 mL Urea (25 g/L)	1 mL Urea (25 g/L)
pH	8.1 ± 0.02	8.2 ± 0.02
Add to mixture of organic and inorganic components	0.9 mL CaCl_2_·2H_2_O (22.2 g/L)	1 mL CaCl_2_·2H_2_O (22.2 g/L)
	0.1 g BSA	0.18 g BSA
		1.5 g Bile salt

After initial pH measurements, each emulsion (6 mL) was added to the digestion juice (35 mL) held in the digestion cell and hydrolyzed upon stirring at 150 rpm and 37°C. The free fatty acids (FFAs) produced upon hydrolysis were quantified by titration with 0.05 M NaOH to the initial pH. The added volume of 0.05 M NaOH was recorded at 1 min intervals for 30 min. Hydrolysis experiments were performed in duplicate. Given that the pancreatic lipase-catalyzed hydrolysis of soybean oil (a triacylglycerol) generally releases two FFA equivalents, the percentage of FFA release from the emulsion (%FFA) was calculated as


$$\text{Released}\;\text{FFA}\;\left(\%\right)=\frac{\;V_{\text{NaOH}\;\left(t\right)}\times M_{\text{NaOH}}\times {\text{Mw}}_{\text{lipid}}}{2\times W_{\text{lipid}}}\times 100,$$

where *V*_NaOH(*t*)_ is the volume of the NaOH solution (L) consumed at time *t*, *M*_NaOH_ is the molarity of the NaOH solution (0.05 M), *Mw*_lipid_ is the molecular weight of the emulsified lipid, i.e., soybean oil (g/mol), and *W*_lipid_ is the weight of the emulsified soybean oil (g).

The initial rate was calculated as described below to compare the hydrolysis kinetics in the initial 5 min period.


$$\text{Initial rate}\;\left(\text{mM}/s\right)=\frac{\;\text{FFA}\;\text{released during}\;5\;\min\;\left(\mu \text{mol}\right)}{\text{Total volume}\;\left(35\;\text{mL}+V_{\text{NaOH}\;\left(t\right)}\right)}÷300\;\left(s\right)$$

$$\text{Released}\;\text{FFA}\;\left(\mu \text{mol}\right)=V_{\text{NaOH}\left(t\right)}\times M_{\text{NaOH}}\times 1000$$

### 2.11 Statistical analysis

Data were presented as the means ± standard deviations of two or more replicates. Analysis of variance was performed using the IBM SPSS Statistics software (ver. 26; IBM Corp. Armonk, NY, United States). The statistical significance of the differences between the experimental means was determined by Duncan’s multiple range test at *p* < 0.05.

## 3 Results

### 3.1 Synthesis and purification of DMPC

**Figure 1:**
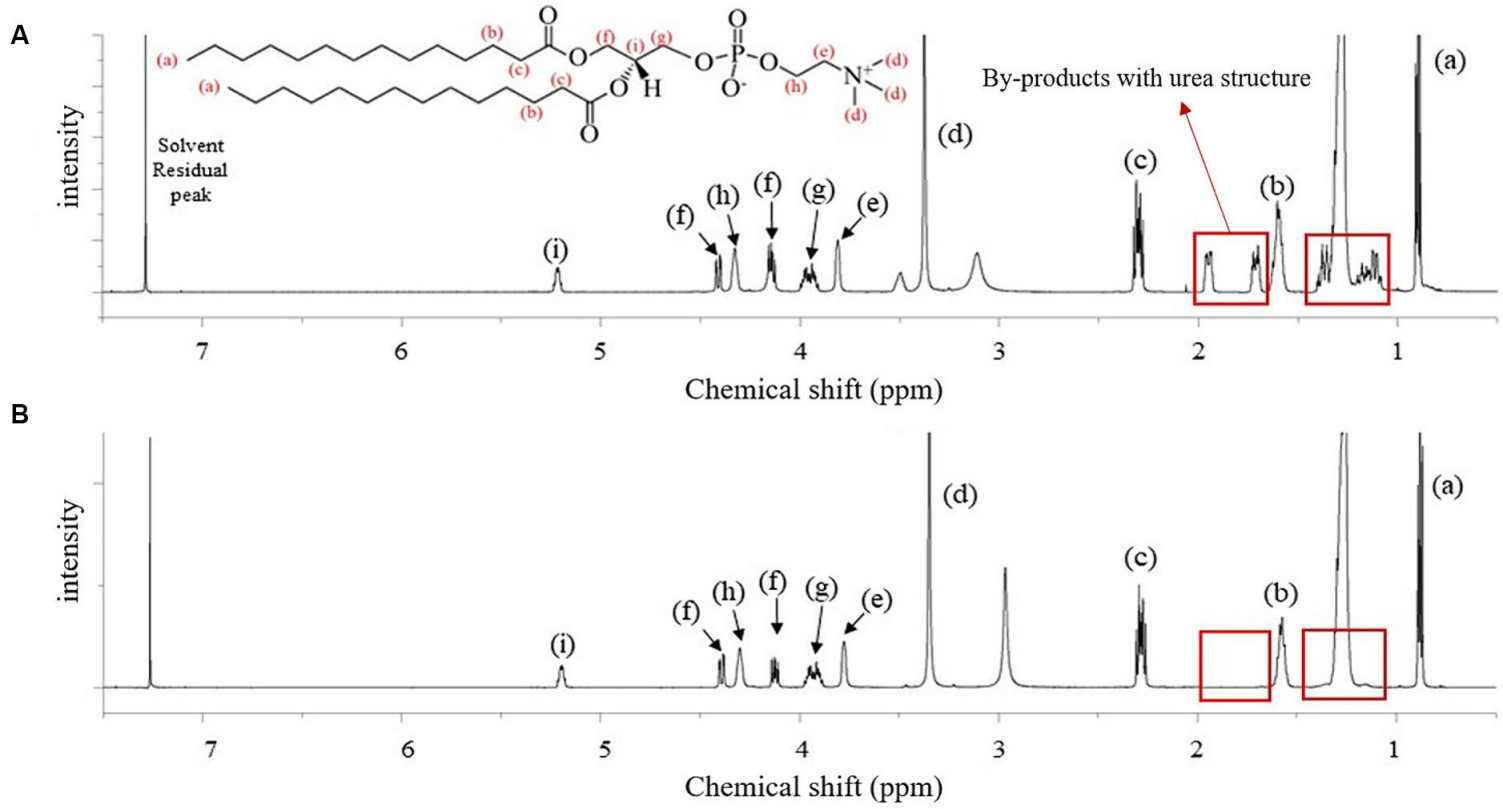
^1^H-NMR spectra of the reactant before and after purification for the synthesis of 1,2-dimyristoyl-sn-glycero-3-phosphocholine (DMPC). **(A)** Reactant after the Steglich esterification reaction; **(B)** finally purified DMPC after acetone recrystallization. Red boxes highlight the by-products with urea structure from the reaction.

Figure 1 shows the spectrum of the crude DMPC obtained immediately after the Steglich esterification (Figure 1A) and that of the final DMPC recrystallized from acetone (Figure 1B). The seven characteristics ^1^H NMR peaks of the phospholipid structure were located between 3.37 and 5.23 ppm. In particular, the methylene group (-CH_2_) at position *sn*-1 and the methine group (-CH) at the position *sn*-2 were represented by peaks (f) and (i) at 4.13–4.42 and 5.20 ppm, respectively (24–26). Thus, ^1^H NMR analysis revealed the presence of fatty acid residues at the *sn*-1 and *sn*-2 positions of the GPC backbone, confirming the successful synthesis of DMPC because only myristic acid as fatty acid was used as a reaction substrate. The ^1^H NMR peaks of urea-type byproducts (e.g., DCU and dicyclohexylacylurea, DCAU) at 1.06–1.4 and 1.70–1.96 ppm (27) were observed in the spectrum of the crude product (Figure 1A) but not in that of the final DMPC (Figure 1B).

**Figure 2:**
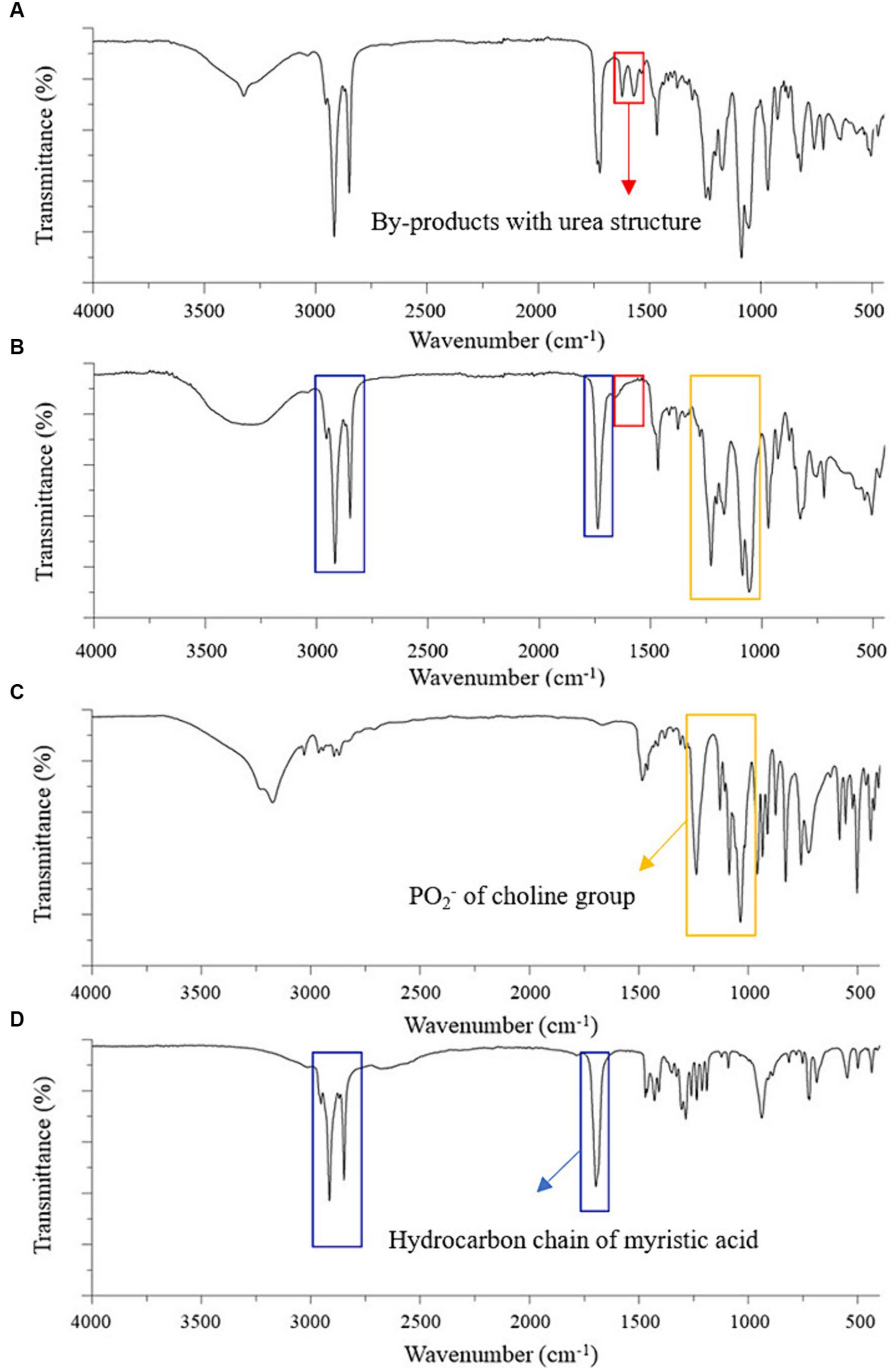
FTIR spectra of the partially purified DMPC, finally purified DMPC, and reaction substrates for DMPC synthesis. **(A)** Partially purified DMPC after ethyl acetate crystallization; **(B)** finally purified DMPC after acetone recrystallization; **(C)** Sn-glycero-3-phosphocholine (GPC); **(D)** Myristic acid. Red boxes highlight by-products with urea structure; yellow boxes highlight PO_2_^−^ of choline group; blue boxes highlight hydrocarbon chain of myristic acid.

The amide I (C=O) and amide II (CO–NH) peaks of the urea-type byproducts at 1,624 and 1,571 cm^−1^ were observed in the FTIR spectrum of the product recrystallized from ethyl acetate (Figure 2A) but not in that of the final DMPC recrystallized from acetone (Figure 2B), which suggested efficient byproduct removal in the latter case (27–29). The spectrum of the final DMPC (Figure 2B) featured the peaks of the choline PO_2_^−^ of choline residue of GPC (1,056–1,229 cm^−1^) and hydrocarbon chain of myristic acid (1,467–1,700 and 2,848–2,914 cm^−1^). The FTIR spectra of the reaction substrates, namely GPC and myristic acid, are provided in Figures 2C,D, respectively (30–33).

**Figure 3:**
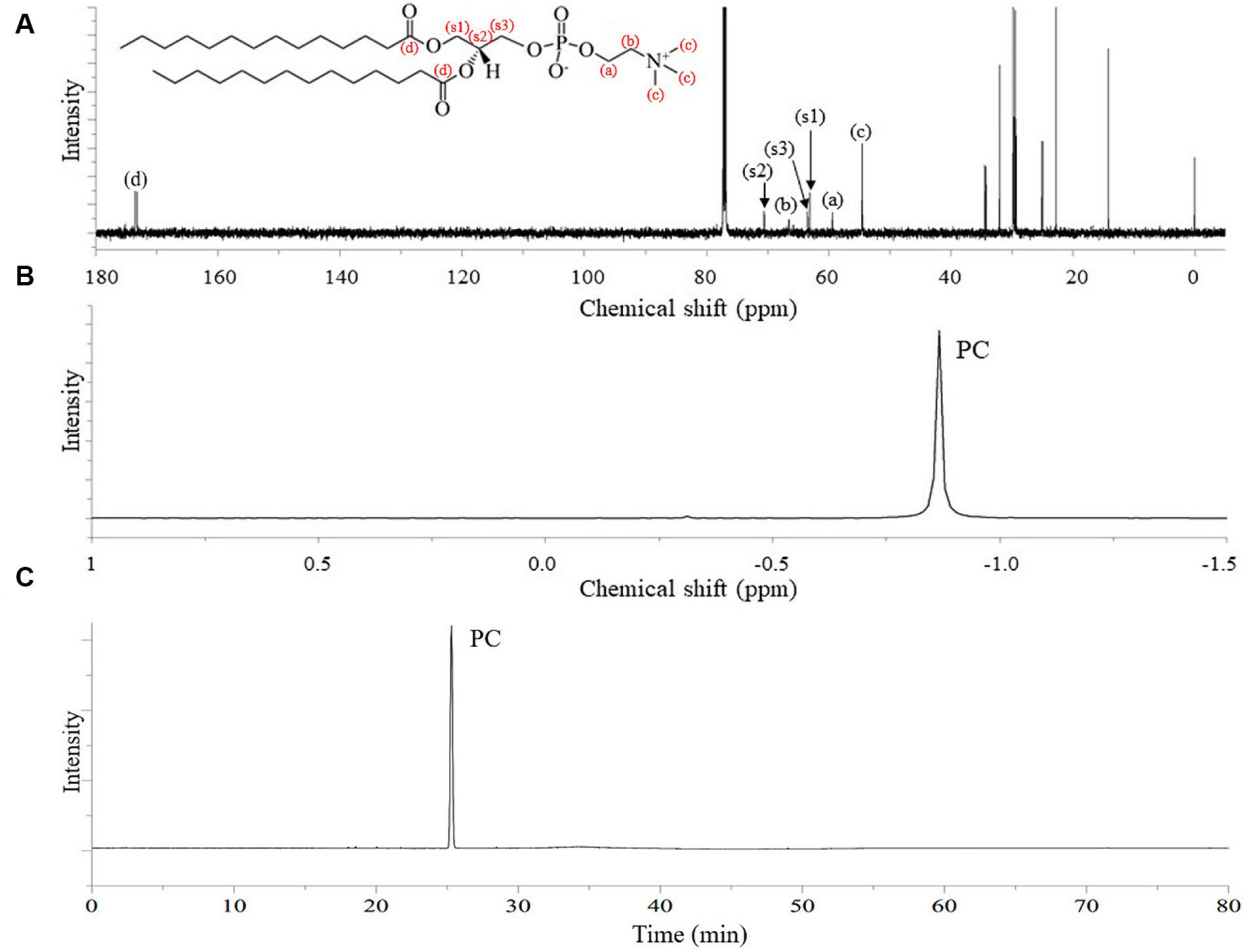
^13^C-NMR and ^31^P-NMR spectra, and HPLC-ELSD chromatogram of finally purified DMPC. **(A)** ^13^C-NMR spectrum; **(B)** ^31^P-NMR spectrum; **(C)** HPLC-ELSD chromatogram.

**Figure 4:**
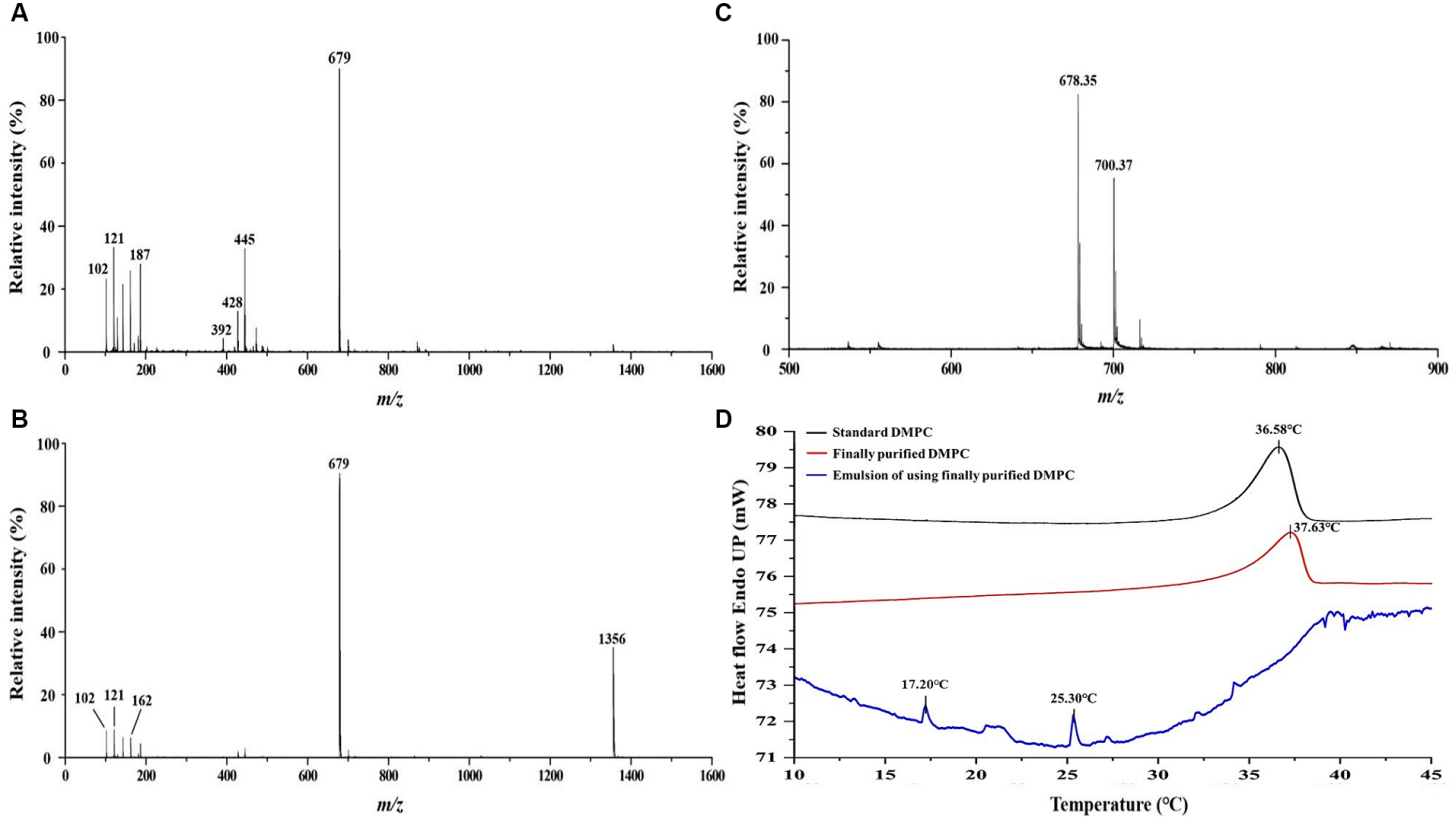
Comparative analysis of DMPC standard and finally purified DMPC using UPLC-MS/MS, MALDI-TOF MS, and DSC. **(A)** UPLC-MS/MS spectrum of DMPC standard; **(B)** UPLC-MS/MS spectrum of finally purified DMPC; **(C)** MALDI-TOF mass spectrum of finally purified DMPC; **(D)** DSC curves of finally purified DMPC, standard DMPC, and the soybean oil-in-water emulsion with DMPC.

The ^13^C NMR spectrum of the final DMPC recrystallized from acetone (Figure 3A) featured the peaks of the hydrocarbon chain of myristic acid residues at positions *sn*-1 and *sn*-2 (14.12–34.36 ppm) and those of glycerol and choline backbones (54.46–70.56 ppm). The corresponding ^31^P NMR spectrum featured the single peak of PC at −0.87 ppm (Figure 3B) and HPLC-ELSD analysis confirmed the purity of the final DMPC was 96% (area%) (Figure 3C). Figure 4 presents the UPLC-MS/MS, MALDI-TOF, and DSC data of the final DMPC and the UPLC-MS/MS and DSC data of the DMPC standard. The UPLC-MS/MS and MALDI-TOF spectra of the final DMPC featured [M + H]^+^ peaks at *m*/*z* 679 and 678.35, respectively, which closely matched the values obtained for the DMPC standard (*m*/*z* 679) and DMPC synthesized in a previous study (*m*/*z* 678.6) (34). Meanwhile, the peaks at *m*/*z* 1,356 in the UPLC-MS/MS (Figure 4B) and 700.37 and in the MALDI-TOF (Figure 4C) spectra of the final DMPC were identified as [2 M + H]^+^ and [M + Na]^+^, respectively. DSC analysis revealed the polymorphic phase behavior of DMPC (Figure 4D) that the *T*_m_ of the final DMPC (37.63°C) was similar to that of the DMPC standard (36.58°C), indicating comparable purity levels. *T*_m_ is a melt transition temperature at which DMPC transitions phase from the gel-ordered state with tightly packed fatty acids to the liquid-crystalline state with disordered fatty acids. This *T*_m_ value of DMPC was expected to decrease upon the incorporation of DMPC into liposomes or hydrated aqueous dispersions, and in the soybean oil-in-water emulsion emulsified with the final DMPC, the *T*_m_ was observed at 25.30°C (Figure 4D). The *T*_m_ of DMPC liposomes by DSC was previously reported as 24.0–24.5°C (35, 36).

### 3.2 Emulsions

#### 3.2.1 Particle size analysis

**Figure 5:**
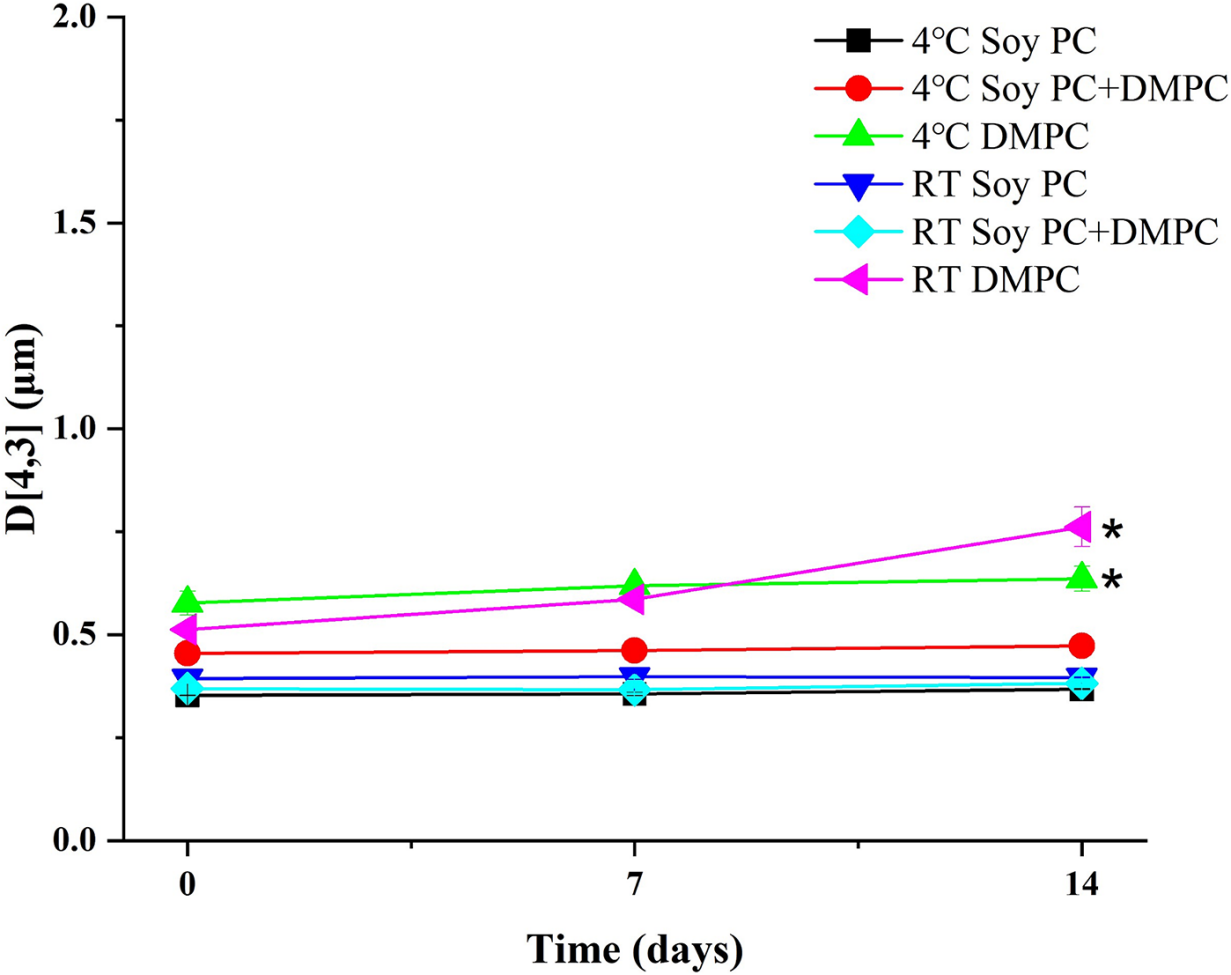
Volume-weighted mean droplet size (*D*[4, 3]) of soy PC, soy PC + DMPC, and DMPC emulsions stored at room temperature and 4°C for 14 days. * Means on the graph are significantly different during storage time by Duncan’s multiple range test at *p* < 0.05.

**Figure 6:**
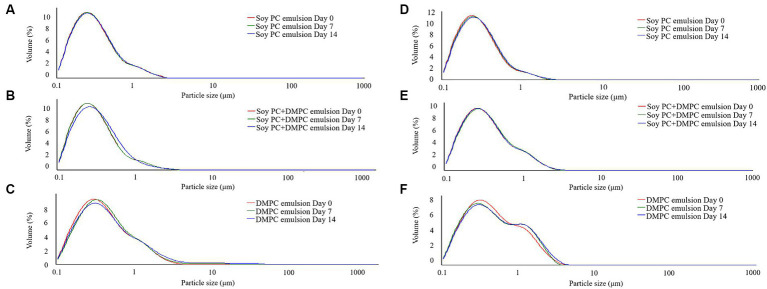
Particle size distribution of emulsions during stored at room temperature and 4°C for 14 days. **(A–C)** Room temperature storage; **(D–F)** 4°C storage.

**Figure 7:**
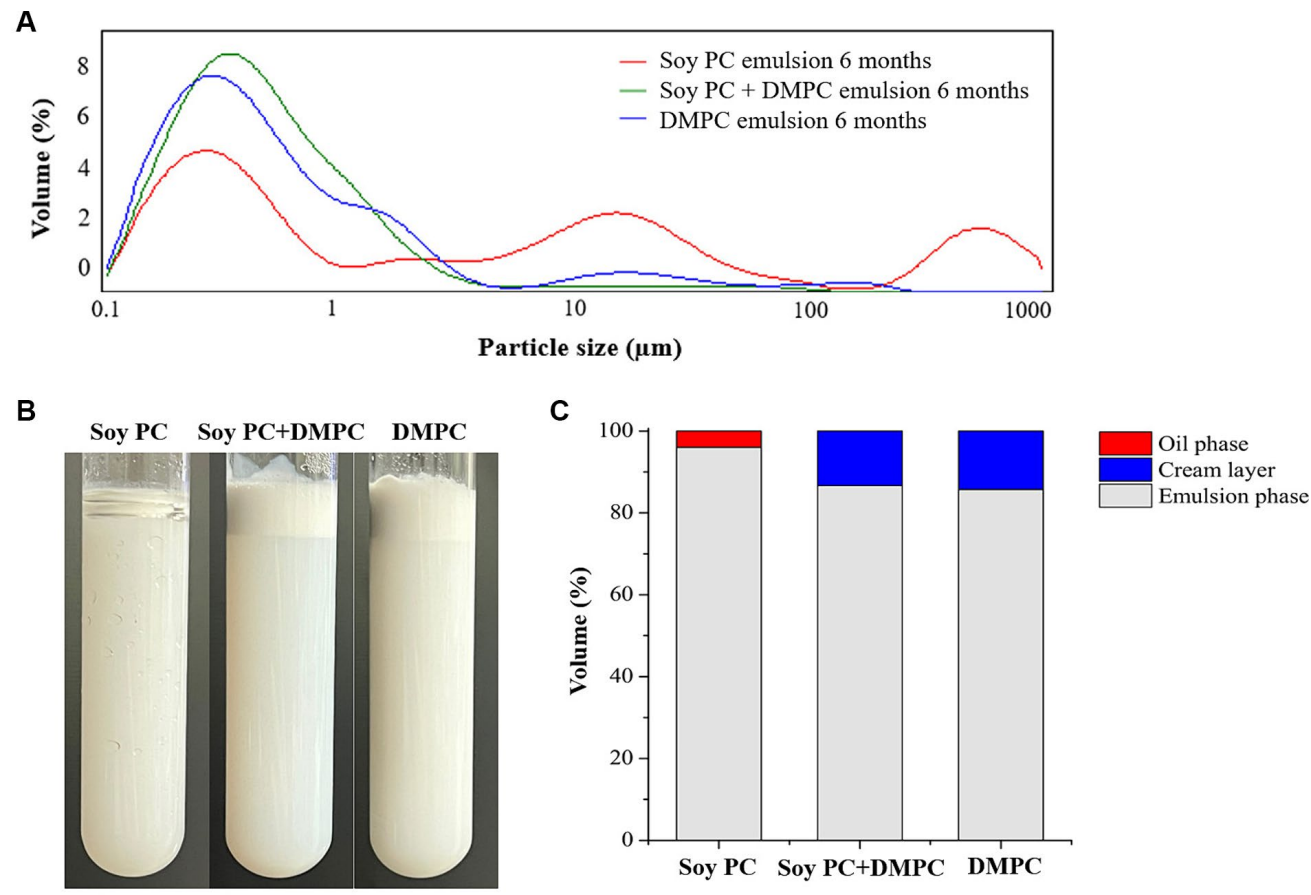
Change in soy PC emulsion after 6 months, soy PC + DMPC emulsion after 6 months, and DMPC emulsion after 6 months of storage at 4°C. **(A)** Particle size distribution; **(B)** appearance; **(C)** volume ratio of oil phase, cream layer, and emulsion phase.

Droplet size is an indicator of emulsion stability, because interfacial tension minimization via a decrease in the droplet surface area/volume ratio is thermodynamically favored (37). After 14 days of storage at 4°C and RT, the *D*[4, 3] of all emulsions were similar ranging from 0.3–0.6 μm to 0.3–0.8 μm, even though there was a statistical difference in *D*[4, 3] values for DMPC emulsion on day 14 regardless of the storage temperatures. Notedly, the *D*[4, 3] value of DMPC emulsion increased from 0.5 μm at Day 0 to 0.8 μm at Day 14 when stored at RT (Figure 5). Further, storage for 14 days had no significant change in the particle size distribution (PSD) (Figures 6A–E), except for the DMPC emulsion stored at 4°C. The size at 90% [*d*(0.9)] of the DMPC emulsion stored at 4°C gradually increased from 1.3 μm on Day 0 to 1.4 μm on Day 14, exhibiting a bimodal character where smaller particles decreased and larger particles increased over time (Figure 6F). This observation suggests that the stability of the DMPC emulsion at 4°C was lower compared to the other emulsions, at least for the duration of this 14 day study. Meanwhile, the soy PC emulsion after 6 months of storage at 4°C contained 10–1,000 μm particles that were absent on day 0, and the sizes at 50% [*d*(0.5)] and *d*(0.9) of the PSD were 2.3 and 395 μm, respectively, indicating a highly unstable state (Figure 7A). On the contrary, the *d*(0.5) and *d*(0.9) values of soy PC + DMPC and DMPC emulsions were 0.42–0.43 and 1.4–2.6 μm, respectively, indicating stabilities higher than those of the soy PC emulsion at 4°C. Visual examination indicated that the soy PC emulsion exhibited the worst long-term shelf-life, with a separated oil phase accounting for approximately 4% of the total emulsion height (Figures 7B,C). However, soy PC + DMPC and DMPC emulsions exhibited a cream layer of approximately 13–14%, without any observable separated oil phase.

#### 3.2.2 Zeta potential measurements

**Figure 8:**
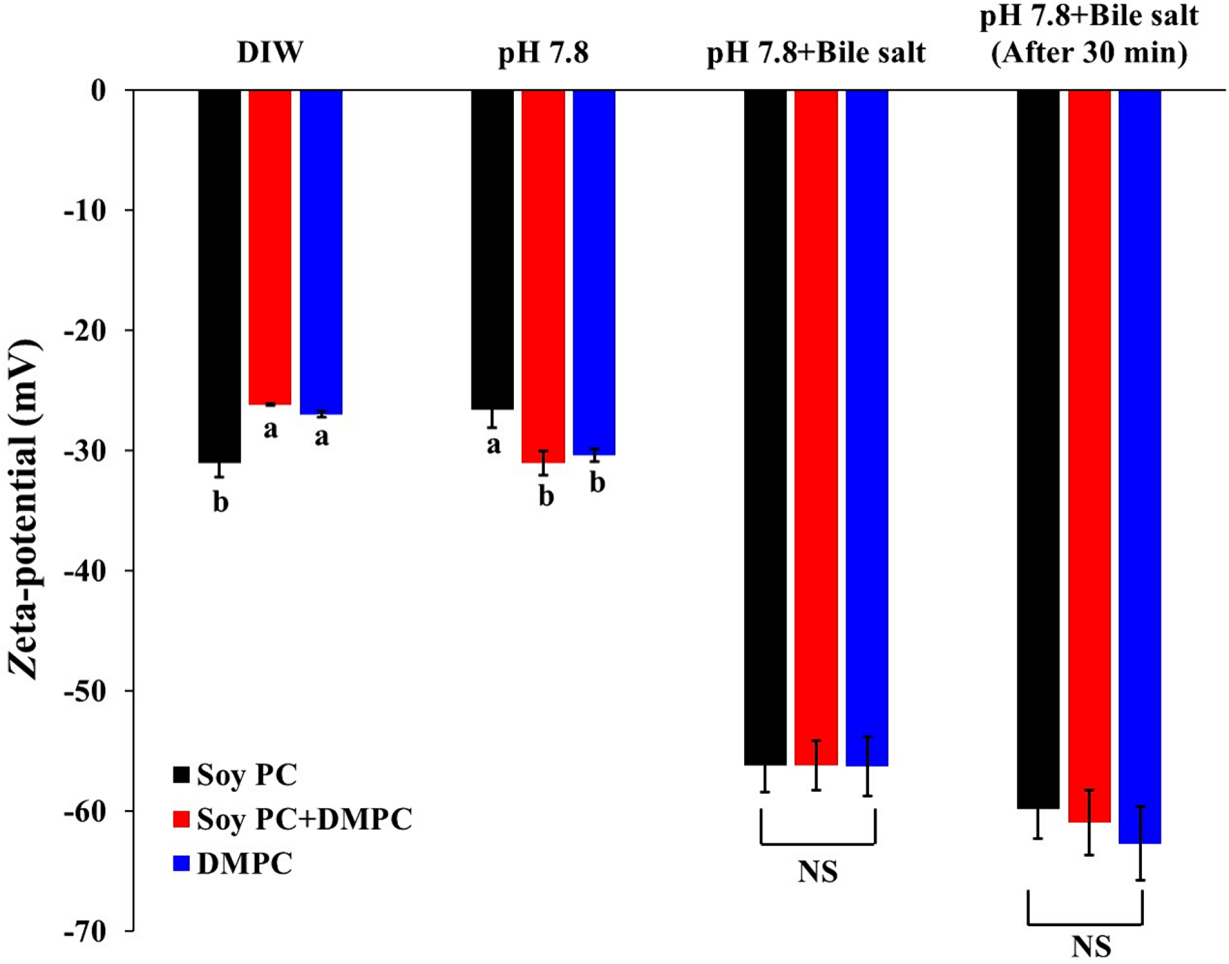
Zeta-potential of emulsion droplets diluted with deionized water (DIW), pH 7.8 buffer, pH 7.8 buffer with bile salts, and pH 7.8 buffer with bile salts after 30 min. The measurements were performed at a constant temperature of 37°C, with the concentration of bile salts mimicking that used in the *in vitro* digestion model. Data are presented as means with standard deviations. ^a,b^ Means on the graph are significantly different by Duncan’s multiple range test at *p* < 0.05. NS, not significant.

The zeta potentials measured after dilution with deionized water were −31.1 mV (soy PC), −26.2 mV (soy PC + DMPC), and −27.0 mV (DMPC) pH 5.8 (Figure 8). Zeta potential measurements were also performed in a bile salt-containing environment, simulating that in the small intestine (37°C and pH 7.8). Bile salts are biosurfactants capable of facilitating the penetration and displacement of other emulsifiers adsorbed on the oil droplet-water interface. Given the difference in *T*_m_ between soy PC and DMPC, the three emulsions were expected to have different droplet-surface phases and, hence, different digestion behaviors.

The zeta potentials of the emulsions diluted with the pH 7.8 buffer were −26.6 mV (soy PC), −31.0 mV (soy PC + DMPC), and −30.4 mV (DMPC). Since the pKa1 (phosphate group’s pKa) of PC is around pH 2 and pKa2 (choline group’s pKa) is above pH 13, the PC head group is expected to be in a zwitterionic form at both pH 5.8 and 7.8, resulting in a small difference (less than 5 mV) in zeta potential between the two conditions. The zeta potentials of the emulsions diluted with the pH 7.8 buffer decreased to −56.2 mV (soy PC), −56.2 mV (soy PC + DMPC), and −56.3 mV (DMPC) immediately after the addition of bile salts (Figure 8). The change in the zeta potential due to bile salt addition [25.2 mV (for soy PC + DMPC) –29.6 mV (for soy PC)] did not significantly vary across the emulsions. However, 30 min after bile salt addition, the zeta potential increased in magnitude by 3.6 to 6.4 mV compared to immediately after addition. This result indicated that the phase variations on the droplet surface above *T*_m_ (37°C) were insufficient to alter the penetration behavior of bile salts, and the adsorption of these salts on the droplet surface in soy PC, soy PC + DMPC, and DMPC emulsions was considered to be rapid. To assess the effects of the bile salt adsorption behavior on the degree of lipolysis, we performed simulated intestinal digestion experiments.

#### 3.2.3 *In vitro* pH-stat digestion

**Figure 9:**
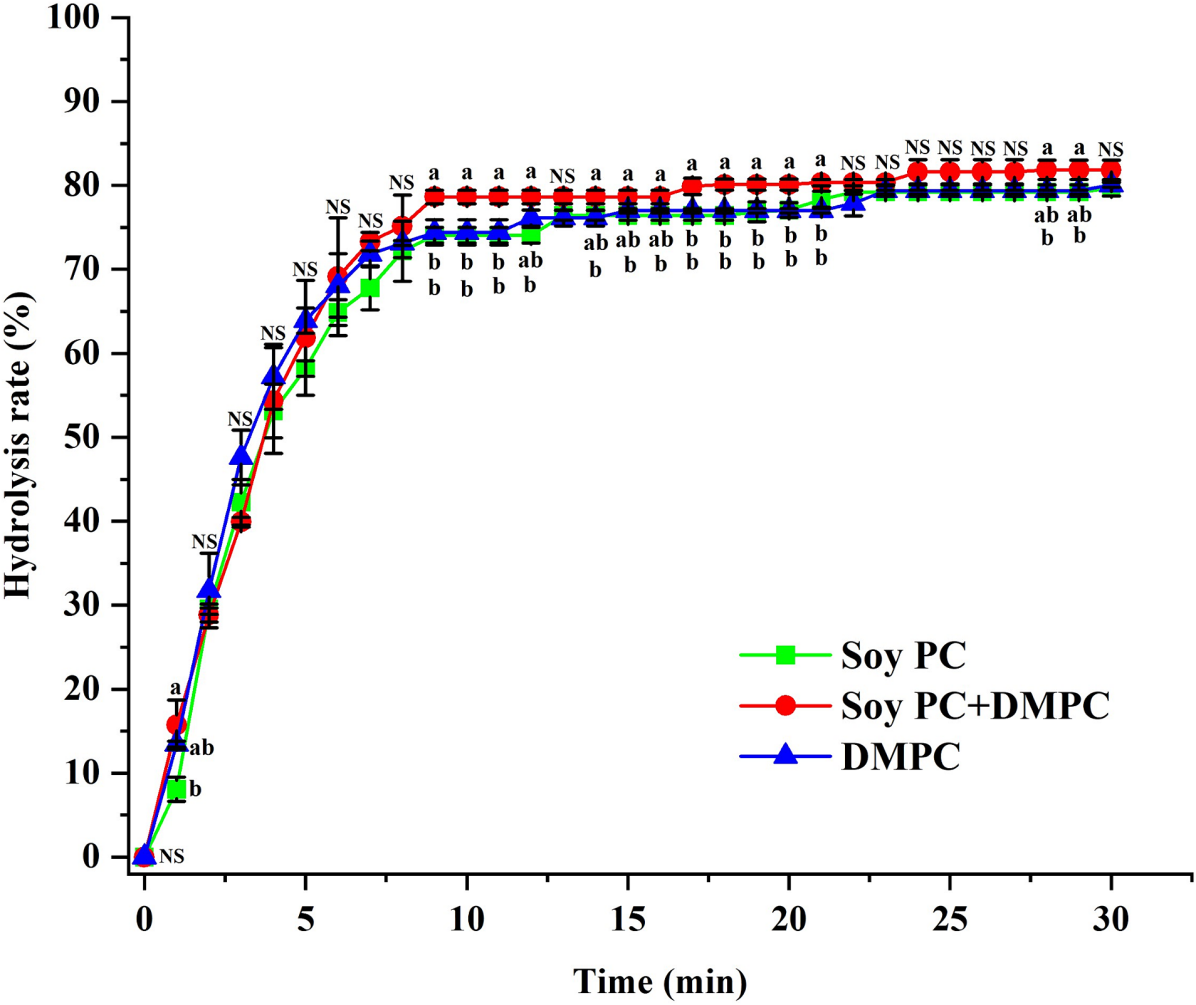
FFA release (%) from soy PC, soy PC + DMPC, and DMPC emulsions for 30 min of *in vitro* digestion simulating the small intestine. A mixture of bile juice and duodenal juice was used as a digestion fluid, along with pancreatic lipase and pancreatin as lipolytic enzymes. The digestion process was performed at 37°C. ^a,b^ Means on the graph are significantly different by Duncan’s multiple range test at *p* < 0.05. NS, not significant.

The initial *in vitro* digestion rate did not significantly vary across the emulsions (*p* > 0.05), showing 0.031, 0.033, and 0.033 mM/s for soy PC, soy PC + DMPC, and DMPC, respectively (Table 2). The released FFA (%) after 30 min of *in vitro* digestion also showed no significant variation (*p* > 0.05), equaling 79.64, 81.84, and 80.06% for soy PC, soy PC + DMPC, and DMPC, respectively (Table 2). The three emulsions exhibited highly similar FFA release profiles, with most of the hydrolysis occurring within 10 min (Figure 9).

**Table 2:** Initial digestion rate (mM/s) and lipolysis rates (as released FFA%) after 30 min of *in vitro* digestion of soy PC, soy PC + DMPC, and DMPC emulsions.

	Initial rate (mM/s)	Lipolysis rate (%) after 30 min of digestion
Soy PC emulsion	0.031 ± 0.000^a^^,^^b^	79.64 ± 0.87^b^
Soy PC + DMPC emulsion	0.033 ± 0.003	81.84 ± 1.16
DMPC emulsion	0.033 ± 0.000	80.06 ± 0.28

a The data are presented as means with standard deviations.

b NS indicated not significant difference between the same columns with Duncan’s multiple range test (*p* > 0.05).

#### 3.2.4 Microstructure observation

**Figure 10:**
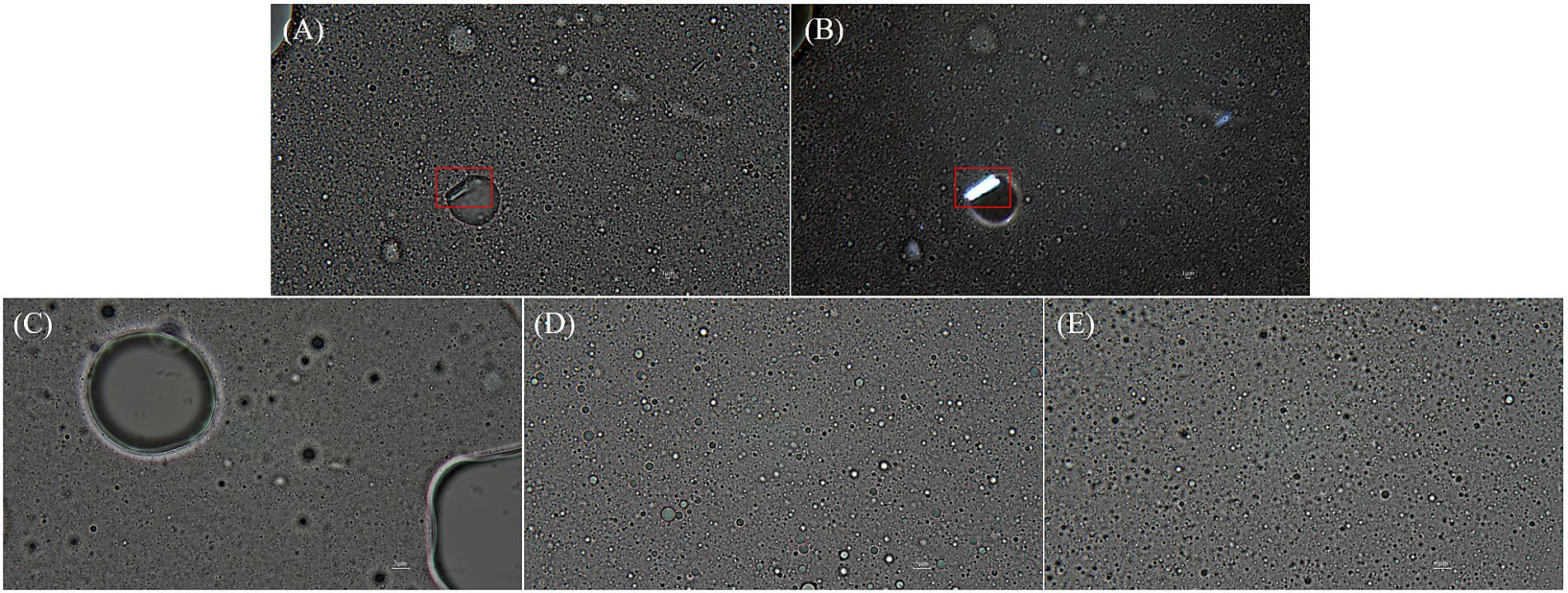
Microscopic images (1,000× magnification) of emulsions after 6 months of storage at 4°C. **(A,B,E)** DMPC emulsion; **(C)** Soy PC emulsion; **(D)** Soy PC + DMPC emulsion. Images were taken using: **(A,C–E)** optical microscope; **(B)** polarized light microscope. Red boxes highlight polygonal crystal structures of DMPC.

After 6 months of storage, oil droplets with polygonal crystals on their surface were observed for the soy PC + DMPC and DMPC emulsions but not for the soy PC emulsion. Compared with the soy PC + DMPC emulsion, the DMPC emulsion contained more oil droplets with polygonal crystals, which appeared to be similar to DMPC crystals in terms of appearance and light transmission (Figures 10A,B). The soy PC + DMPC (Figure 10D) and DMPC (Figure 10E) emulsions displayed a stable state with a consistently small droplet size, whereas the soy PC (Figure 10C) system contained very large droplets (in agreement with Figure 7A) and the number of small droplets in the dispersed phase decreased.

## 4 Discussion

Given its high polarity, GPC did not readily participate in the Steglich esterification unless thoroughly dispersed in the reaction medium, i.e., chloroform (log *p* = 2.00). Hence, a silica–GPC complex was prepared to facilitate the dispersion of GPC and increase its reactivity. The rapid binding of the activated fatty acids to GPC is crucial for the Steglich esterification, as the DCAU otherwise produced as a byproduct affects the product yield and purity. To resolve this problem, we used DMAP to accelerate the DCC-mediated esterification of carboxylic acids, prevent DCAU formation, and ensure the preferential formation of DCU as a byproduct. The poorly soluble DCU formed short, spiky crystals when the product was left to stand after dissolution in an organic solvent. Hence, DCU was partially removed through filtration prior to sequential crystallization (38–40), while the basic DMAP was effectively removed using a 0.25 N HCl—methanol solution (41).

The isolation and purification of DMPC by sequential crystallization relied on the differences in the solubility of this compound in a specific solvent. After the vacuum concentration of the chloroform layer, the unreacted substrate (myristic acid), coupling agent (DCC), and byproducts (e.g., fatty acid anhydrides) were removed using ethyl acetate, indicating that DMPC has low solubility in ethyl acetate. To maximize this solubility difference, we adjusted the ethyl acetate temperature to 50°C to dissolve both DMPC and the undesired species, and then cooled the solution to 4°C to precipitate DMPC. The crystallization from ethyl acetate was repeated several times to remove the major (urea-type) byproducts; however, the complete removal of these species was not possible. Thus, recrystallization from acetone was performed as the last purification step. DMPC crystallizes together with water molecules in a water-containing solution, forming a bilayer structure due to strong hydrogen bonding in the polar portions of DMPC. Therefore, in DMPC crystals, hydrophobic acyl chains are arranged inward, and hydrophilic head groups are oriented toward the surface. Crystal nuclei formed when the solution temperature decreased to <14°C and appeared as a flocculent precipitate. Subsequent crystal growth occurred at 0–22°C (42). Given the chain-alignment collapse and melting observed above the *T*_m_ (37.63°C for pure DMPC), recrystallization from acetone was performed at −6°C and efficiently removed impurities such as DCU and DCAU, as confirmed by ^1^H NMR, FTIR, and HPLC-ELSD analyses.

The results of the emulsion stability evaluation suggested that the phase transition of DMPC was the main factor responsible for the observed stability differences. In the liposomal dispersion of DMPC, the lamellar-structured DMPC experiences two phase transitions, namely the pre-transition (gel → ripple gel phase) and the main phase transition (ripple gel phase → liquid-crystalline phase). The pre-transition occurs when the temperature increases to 15°C (*T*_p_) and corresponds to a change from a well-ordered gel phase to a ripple gel phase, in which the acyl chains in the bilayer move less cooperatively and form periodic ripples. Further the main phase transition occurs at the temperature of 23.4°C (*T*_m_) in the DMPC liposomes, the ripple gel phase changes to a liquid-crystalline phase with disordered acyl chains (43–45). In the DMPC emulsion with soybean oil, the *T*_p_ and *T*_m_ of the DMPC emulsion were 17.20°C and 25.30°C, respectively (Figure 4D), which are similar to those of DMPC liposomes, and it is suggested that the phase transitions influence the stability of the DMPC emulsion. In contrast, the *T*_m_ of soy PC ranges from −48.5 to −13.1°C, which leads to the gel → liquid-crystalline phase transition (12). Therefore, the phase transition of soy-PC and/or DMPC affects the stability of emulsions with soybean oil using them as emulsifiers.

Based on the phase transition characteristics, we predicted the phases present on the droplet surface (o/w interface) at 4°C and RT. For the soy PC emulsion, the phase was predicted to be liquid-crystalline regardless of temperature, whereas for the soy PC + DMPC emulsion, a coexistence of liquid-crystalline and gel phases was expected at 4°C and a liquid-crystalline phase at RT. On the contrary, the DMPC emulsion was predicted to contain the gel phase at 4°C and liquid-crystalline phase at RT. As shown in Figure 5, the *D*[4, 3] values of all three emulsions exhibited minimal change between Day 0 and Day 7, regardless of the temperature, indicating that the emulsions maintained high stability for 7 days. However, while the other emulsions maintained consistent *D*[4, 3] values and PSDs throughout the 14 days, the DMPC emulsion demonstrated a significant increase in *D*[4, 3] values at both 4°C and RT on Day 14, suggesting a decrease in stability. Notably, the DMPC emulsion developed a bimodal PSD starting from Day 7, exhibiting lower stability compared to the other emulsions (Figure 6). When DMPC emulsion stored at 4°C (i.e., lower than *T*_m_ of DMPC), a corrugated surface probably formed by phenomena (e.g., transition to gel phase on the droplet surface, shrinkage due to increase in the density of the internal oil, etc.) led to partial coalescence with adjacent droplets (46). The aggregation caused by this partial coalescence was held responsible for the increase in the ratio of large droplets in the DMPC emulsion and shift of its PSD toward bimodality. In contrast, no significant changes were observed in the PSD of soy PC and soy PC + DMPC emulsions stored at 4°C for 14 days, which were predicted to be either a liquid-crystalline phase or a coexistence of liquid-crystalline and gel phases. This implies that the droplet surface, containing more than half of soy PC, was resistant to partial coalescence, indicating the stability of the emulsions over 14 days.

The soy PC + DMPC and DMPC emulsions were notably more stable than the soy PC emulsion after 6 months of storage at 4°C (Figure 7). This finding was ascribed to the structure (e.g., saturation degree and length) of the acyl chains bound to the PC head group and corresponding implications for the physicochemical properties of the droplet surface, which affected emulsion stability in a complex way. Given the linear structure of myristic acid residues (i.e., the absence of kinks due to *cis* double bonds), DMPC may exist in a gel phase at 4°C, forming a packed and low fluidity droplet surface (43). DMPC emulsions exhibited a little instability during short-term storage (up to 14 days) at 4°C. This instability was attributed to partial coalescence and other factors affecting the gel phase on the droplet surface. Conversely, DMPC emulsions forming a robust gel phase exhibited superior stability over a longer storage period (6 months) at 4°C. This advantage stemmed from their ability to prevent oil-off instability that is observed in soy PC emulsion. In addition, the hydrophilic–lipophilic value of PCs increases with the decreasing length of the acyl chain. Therefore, DMPC, which contained a 14 carbon myristic acid residue, formed a more stable o/w emulsion than soy PC, which featured longer (16–18 carbon) acyl residues (20).

Zeta-potential describes the electrical potential between electrical attraction and repulsion at the emulsion droplet surface (47). Emulsions diluted with deionized water at pH 5.8 and with buffer at pH 7.8 (Figure 8) had similar negative zeta potentials, which indicated that the contribution of Coulombic repulsion to storability did not vary across the emulsions. This result was explained by the identical charged head groups of soy PC and DMPC.

To examine the changes in the penetration and displacement of bile salts according to the characteristics of the droplet surface, we recorded the zeta potentials of the emulsions under the conditions of simulated small-intestinal digestion. Bile salts, in the form of sodium carboxylates, improve lipolysis on the droplet surface, and the carboxylate ions formed upon the dissociation of sodium generate a stronger negative charge than PC. Hence, the displacement of PC with bile salts on the droplet surface results in a larger negative charge, implying that change of zeta potential is an indicator of how much bile salts have replaced the existing PC (48–51). According to a previous study, the droplet surface of 1,2-dioleoyl-*sn*-glycero-3-phosphocholine and DMPC-based emulsions at 37°C was a liquid-crystalline phase with a higher penetrability and capability to bind plasma proteins than the gel phase of DPPC- and DSPC-based emulsions, which resulted in higher lipolysis in plasma (52).

The similarity of zeta potentials after the addition of bile salts implied that at 37°C, these salts were adsorbed on the droplet surface in all three emulsions at similar levels (Figure 8), which agreed with the similarity of the FFA release profiles and hydrolysis rates observed in the *in vitro* digestion experiments (Table 2 and Figure 9). Droplets of emulsion stabilized by PC might exhibit unstable interfaces when exposed to temperatures exceeding their *T*_m_. This destabilization triggered by the phase transition of the droplet surface might promote the interaction between the intra-droplet oil (e.g., triacylglycerols, diacylglycerols) and the lipase enzyme, facilitating rapid FFA release and consequently augmenting the initial and overall rate of lipolysis. The DMPC emulsion has a transition temperature of 25.3°C (Figure 4D), while soy PC has the transition temperature below 0°C (12), and these temperatures are below 37°C, where bile salts added. The droplet surfaces of all three emulsions are in the liquid-crystalline phase with high fluidity and permeability at 37°C. Therefore, the droplet surface among the three emulsions would not affect a significant impact on Zeta-potential of emulsion droplets under pH 7.8 buffer with bile salts for *in vitro* digestion study.

## 5 Conclusion

A cost-effective method for synthesizing high-purity DMPC through Steglich esterification followed by sequential crystallization was developed. Given its high cost, DMPC is presently utilized in limited quantities, primarily in the pharmaceutical industry. However, our research facilitates more economical DMPC production, thereby broadening its potential as a food ingredient. Additionally, storage stability tests conducted on soy PC, soy PC + DMPC, and DMPC emulsions revealed that, over six months of storage at 4°C, emulsions containing DMPC exhibited significantly smaller PSD changes compared to those containing soy PC. Consequently, our results indicate that DMPC holds promise as an emulsifier with wide-ranging applications in the food industry.

## Data availability statement

The original contributions presented in the study are included in the article/Supplementary material, further inquiries can be directed to the corresponding author.

## Author contributions

S-YK: Conceptualization, Formal analysis, Investigation, Methodology, Visualization, Writing – original draft, Writing – review & editing. Y-LP: Conceptualization, Formal analysis, Investigation, Methodology, Visualization, Writing – original draft. H-EJ: Methodology, Writing – original draft. H-SL: Investigation, Writing – original draft. H-JC: Formal analysis, Methodology, Visualization, Writing – review & editing. G-HB: Investigation, Writing – original draft. J-HL: Conceptualization, Funding acquisition, Methodology, Project administration, Resources, Supervision, Visualization, Writing – original draft, Writing – review & editing.

## Glossary

DCAU: dicyclohexylacylurea
DCC: *N*,*N*′-dicyclohexylcarbodiimide
DCU: dicyclohexylurea
DMAP: 4-dimethylaminopyridine
DMPC: 1,2-dimyristoyl-*sn*-glycero-3-phosphocholine
DPPC: 1,2-dipalmitoyl-*sn*-glycero-3-phosphocholine
DSC: differential scanning calorimetry
DSPC: 1,2-distearoyl-*sn*-glycero-3-phosphocholine
ELSD: evaporative light scattering detector
FFA: free fatty acid
FTIR: Fourier transform infrared
GPC: *sn*-glycero-3-phosphocholine
HPLC: high-performance liquid chromatography
L*_α_*: liquid-crystalline phase
L*_β_*: gel phase
L*_c_*: subgel or crystalline phase
MALDI-TOF-MS: matrix-assisted laser desorption and ionization time-of-flight mass spectrometry
NMR: nuclear magnetic resonance
o/w: oil-in-water
P*_β_*: ripple gel phase
PC: phosphatidylcholine
PSD: particle size distribution
Soy PC: soybean phosphatidylcholine
UPLC-MS/MS: ultra-performance liquid chromatography–tandem mass spectrometry
w/o: water-in-oil
w/o/w: water-in-oil-in-water

## References

[1] Hu Y Wang L McClements DJ. Design, characterization and digestibility of β-carotene-loaded emulsion system stabilized by whey protein with chitosan and potato starch addition. Food Chem. (2024) 440:138131. doi: 10.1016/j.foodchem.2023.138131, PMID: 38103502

[2] McClements DJ. Crystals and crystallization in oil-in-water emulsions: implications for emulsion-based delivery systems. Adv Colloid Interf Sci. (2012) 174:1–30. doi: 10.1016/j.cis.2012.03.002 22475330

[3] Li J Wang X Zhang T Wang C Huang Z Luo X. A review on phospholipids and their main applications in drug delivery systems. Asian J Pharm Sci. (2015) 10:81–98. doi: 10.1016/j.ajps.2014.09.004

[4] McClements DJ Li Y. Structured emulsion-based delivery systems: controlling the digestion and release of lipophilic food components. Adv Colloid Interf Sci. (2010) 159:213–28. doi: 10.1016/j.cis.2010.06.010 20638649

[5] Troise AD Fogliano V Madadlou A. Tailor it up! How we are rolling towards designing the functionality of emulsions in the mouth and gastrointestinal tract. Curr Opin Food Sci. (2020) 31:126–35. doi: 10.1016/j.cofs.2020.06.002

[6] McClements DJ. Edible nanoemulsions: fabrication, properties, and functional performance. Soft Matter. (2011) 7:2297–316. doi: 10.1039/C0SM00549E

[7] Niu H Wang W Dou Z Chen X Chen X Chen H. Multiscale combined techniques for evaluating emulsion stability: a critical review. Adv Colloid Interf Sci. (2023) 311:102813. doi: 10.1016/j.cis.2022.102813, PMID: 36403408

[8] Berton-Carabin C Schroën K. Towards new food emulsions: designing the interface and beyond. Curr Opin Food Sci. (2019) 27:74–81. doi: 10.1016/j.cofs.2019.06.006

[9] Lordan R Tsoupras A Zabetakis I. Phospholipids of animal and marine origin: structure, function, and anti-inflammatory properties. Molecules. (2017) 22:1964. doi: 10.3390/molecules22111964, PMID: 29135918 PMC6150200

[10] Palacios LE Wang T. Egg-yolk lipid fractionation and lecithin characterization. J Am Oil Chem Soc. (2005) 82:571–8. doi: 10.1007/s11746-005-1111-4

[11] Zhao F Li R Liu Y Chen H. Perspectives on lecithin from egg yolk: extraction, physicochemical properties, modification, and applications. Front Nutr. (2023) 9:1082671. doi: 10.3389/fnut.2022.1082671, PMID: 36687715 PMC9853391

[12] Urade R Okamoto S Yagi T Moriyama T Ogawa T Kito M. Functions of soy phosphatidylcholine in dough and bread supplemented with soy protein. J Food Sci. (2006) 68:1276–82. doi: 10.1111/j.1365-2621.2003.tb09639.x

[13] Holthuis JCM Menon AK. Lipid landscapes and pipelines in membrane homeostasis. Nature. (2014) 510:48–57. doi: 10.1038/nature13474, PMID: 24899304

[14] Ishii F Nii T. Properties of various phospholipid mixtures as emulsifiers or dispersing agents in nanoparticle drug carrier preparations. Colloids Surf B: Biointerfaces. (2005) 41:257–62. doi: 10.1016/j.colsurfb.2004.12.018 15748821

[15] Hörmann K Zimmer A. Drug delivery and drug targeting with parenteral lipid nanoemulsions—a review. J Control Release. (2016) 223:85–98. doi: 10.1016/j.jconrel.2015.12.016, PMID: 26699427

[16] van Hoogevest P Wendel A. The use of natural and synthetic phospholipids as pharmaceutical excipients. Eur J Lipid Sci Technol. (2014) 116:1088–107. doi: 10.1002/ejlt.201400219, PMID: 25400504 PMC4207189

[17] Anderson M Omri A. The effect of different lipid components on the in vitro stability and release kinetics of liposome formulations. Drug Deliv. (2004) 11:33–9. doi: 10.1080/10717540490265243, PMID: 15168789

[18] Bhardwaj U Burgess DJ. Physicochemical properties of extruded and non-extruded liposomes containing the hydrophobic drug dexamethasone. Int J Pharm. (2010) 388:181–9. doi: 10.1016/j.ijpharm.2010.01.003, PMID: 20079409

[19] Leonenko ZV Finot E Ma H Dahms TE Cramb DT. Investigation of temperature-induced phase transitions in DOPC and DPPC phospholipid bilayers using temperature-controlled scanning force microscopy. Biophys J. (2004) 86:3783–93. doi: 10.1529/biophysj.103.036681, PMID: 15189874 PMC1304279

[20] Nii T Ishii F. Properties of various phosphatidylcholines as emulsifiers or dispersing agents in microparticle preparations for drug carriers. Colloids Surf B: Biointerfaces. (2004) 39:57–63. doi: 10.1016/j.colsurfb.2004.08.017 15542341

[21] Ichihara K Iwasaki H Ueda K Takizawa R Naito H Tomosugi M. Synthesis of phosphatidylcholine: an improved method without using the cadmium chloride complex of sn-glycero-3-phosphocholine. Chem Phys Lipids. (2005) 137:94–9. doi: 10.1016/j.chemphyslip.2005.06.001, PMID: 16054615

[22] Melamed P Melamed F. Chronic metabolic acidosis destroys pancreas. JOP J Pancreas. (2014) 15:552–60. doi: 10.6092/1590-8577/2854, PMID: 25435570

[23] Versantvoort CHM Oomen AG Van de Kamp E Rompelberg CJ Sips AJ. Application of an in vitro digestion model in assessing the bioaccessibility of mycotoxins in food. Food Chem Toxicol. (2005) 43:31–40. doi: 10.1016/j.fct.2004.08.007, PMID: 15582193

[24] Wang X Yang D Gan LJ Zhang H Shin JA Park SH. Degree of oxidation depending on the positional distribution of linolenic acid in perilla oil and interesterified products. Food Sci Biotechnol. (2014) 23:1733–40. doi: 10.1007/s10068-014-0237-7

[25] Stevens MM Honerkamp-Smith AR Keller SL. Solubility limits of cholesterol, lanosterol, ergosterol, stigmasterol, and β-sitosterol in electroformed lipid vesicles. Soft Matter. (2010) 6:5882–90. doi: 10.1039/c0sm00373e, PMID: 21731574 PMC3124637

[26] Brunetti E Moerkerke S Wouters J Bartik K Jabin I. A selective calix[6]arene-based fluorescent chemosensor for phosphatidylcholine type lipids. Org Biomol Chem. (2016) 14:10201–7. doi: 10.1039/C6OB01880G, PMID: 27731470

[27] Shang J Liu S Ma X Lu L Deng Y. A new route of CO2 catalytic activation: syntheses of N-substituted carbamates from dialkyl carbonates and polyureas. Green Chem. (2012) 14:2899–906. doi: 10.1039/C2GC36043H

[28] Movasaghi Z Rehman S ur Rehman I. Fourier transform infrared (FTIR) spectroscopy of biological tissues. Appl Spectrosc Rev. (2008) 43:134–79. doi: 10.1080/05704920701829043

[29] Kim JY Chung DW. Study on the synthesis of N,N’-dicyclohexylcarbodiimide from N,N’-dicyclohexylurea. Appl Chem Eng. (2011) 22:319–22. doi: 10.14478/ACE.2011.22.3.319

[30] Derenne A Claessens T Conus C Goormaghtigh E. Infrared spectroscopy of membrane lipids, Roberts GCK, (Ed.), Encyclopedia of biophysics. Berlin, FL: Springer (2013). 1074–1081

[31] Bridelli MG Capelletti R Mora C. Structural features and functional properties of water in model DMPC membranes: thermally stimulated depolarization currents (TSDCs) and Fourier transform infrared (FTIR) studies. J Phys D Appl Phys. (2013) 46:485401–11. doi: 10.1088/0022-3727/46/48/485401

[32] Pereira A Mallya R. Formulation and evaluation of a photoprotectant cream containing Phyllanthus emblica extract- phospholipid complex. J pharmacogn phytochem. (2015) 4:232–40.

[33] Su L Zhang B Huang Y Fan Z Zhao Y. Enhanced cellular uptake of iron oxide nanoparticles modified with 1,2-dimyristoyl-sn-glycero-3-phosphocholine. RSC Adv. (2017) 7:38001–7. doi: 10.1039/C7RA06844A

[34] Hu L Xie G Lan Q Yu Z Hu L Zhu L. Quantitative UPLC-MS/MS to detect DMPC and DPPC applied to paraquat poisoning in cells and serum. Chromatographia. (2022) 85:147–53. doi: 10.1007/s10337-021-04113-z

[35] Adão R Nazmi K Bolscher JGM Bastos M. C- and N-truncated antimicrobial peptides from LFampin 265–284: biophysical versus microbiology results. J Pharm Bioallied Sci. (2011) 3:60–9. doi: 10.4103/0975-7406.76467, PMID: 21430955 PMC3053522

[36] Chen W Duša F Witos J Ruokonen SK Wiedmer S. Determination of the main phase transition temperature of phospholipids by nanoplasmonic sensing. Sci Rep. (2018) 8:14815. doi: 10.1038/s41598-018-33107-5, PMID: 30287903 PMC6172256

[37] Fox CB Anderson RC Dutill TS Goto Y Reed SG Vedvick TS. Monitoring the effects of component structure and source on formulation stability and adjuvant activity of oil-in-water emulsions. Colloids Surf B: Biointerfaces. (2008) 65:98–105. doi: 10.1016/j.colsurfb.2008.03.003, PMID: 18440205

[38] Jordan A Whymark K Sydenham J Sneddon H. A solvent-reagent selection guide for Steglich-type esterification of carboxylic acids. Green Chem. (2021) 23:6405–13. doi: 10.1039/D1GC02251B

[39] Gibson FS Park MS Rapoport H. Bis[[4-(2,2-dimethyl-1,3-dioxolyl)]methyl]- carbodiimide (BDDC) and its application to residue-free esterifications, peptide couplings, and dehydrations. J Am Chem Soc. (1994) 59:7503–7. doi: 10.1021/jo00103a054

[40] Neises B Steglich W. Esterification of carboxylic acids with dicyclohexylcarbodiimide/4-dimethylaminopyridine: tert-butyl ethyl fumarate. Org Synth. (2003) 63:183. doi: 10.1002/0471264180.os063.22

[41] Ihre H Padilla De Jesús OL Fréchet JM. Fast and convenient divergent synthesis of aliphatic ester dendrimers by anhydride coupling. J Am Chem Soc. (2001) 123:5908. doi: 10.1021/ja010524e, PMID: 11414823

[42] Pearson RH Pascher I. The molecular structure of lecithin dihydrate. Nature. (1979) 281:499–501. doi: 10.1038/281499a0, PMID: 492310

[43] Lombardo D Kiselev MA. Methods of liposomes preparation: formation and control factors of versatile nanocarriers for biomedical and nanomedicine application. Pharm. (2022) 14:543. doi: 10.3390/pharmaceutics14030543, PMID: 35335920 PMC8955843

[44] Sharma VK Mamontov E Ohl M Tyagi M. Incorporation of aspirin modulates the dynamical and phase behavior of the phospholipid membrane. Phys Chem Chem Phys. (2017) 19:2514–24. doi: 10.1039/c6cp06202d, PMID: 28058428

[45] Pentak D. Alternative methods of determining phase transition temperatures of phospholipids that constitute liposomes on the example of DPPC and DMPC. Thermochim Acta. (2014) 584:36–44. doi: 10.1016/j.tca.2014.03.020

[46] Nikolai D Slavka T Cholakova D. Surface phase transitions in foams and emulsions. Curr Opin Colloid Interface Sci. (2019) 44:32–47. doi: 10.1016/j.cocis.2019.09.005

[47] Hong IK Kim SI Lee SB. Effects of HLB value on oil-in-water emulsions: droplet size, rheological behavior, zeta-potential, and creaming index. J Ind Eng Chem. (2018) 67:123–31. doi: 10.1016/j.jiec.2018.06.022

[48] Wickham M Garrood M Leney J Wilson PDG Fillery-Travis A. Modification of a phospholipid stabilized emulsion interface by bile salt: effect on pancreatic lipase activity. J Lipid Res. 39:623–32. doi: 10.1016/S0022-2275(20)33300-9 9548594

[49] Yang L Tucker IG Østergaard J. Effects of bile salts on propranolol distribution into liposomes studied by capillary electrophoresis. J Pharm Biomed Anal. (2011) 56:553–9. doi: 10.1016/j.jpba.2011.06.020, PMID: 21784594

[50] Zhang H Lv M Jiang J Cui Z Xia W Binks BP. Conversion of bile salts from inferior emulsifier to efficient smart emulsifier assisted by negatively charged nanoparticles at low concentrations. Chem Sci J. (2021) 12:11845–50. doi: 10.1039/d1sc02596a, PMID: 34659724 PMC8442726

[51] Bellesi FA Martinez MJ Ruiz-Henestrosa VMP Pilosof AM. Comparative behavior of protein or polysaccharide stabilized emulsion under in vitro gastrointestinal conditions. Food Hydrocoll. 52:47–56. doi: 10.1016/j.foodhyd.2015.06.007

[52] Zhang B Zhou X Miao Y Wang X Yang Y Zhang X. Effect of phosphatidylcholine on the stability and lipolysis of nanoemulsion drug delivery systems. Int J Pharm. (2020) 583:119354. doi: 10.1016/j.ijpharm.2020.119354, PMID: 32348799

