# Supplementary figures and images for: High-purity 1,2-dimyristoyl-sn-glycero-3-phosphocholine: synthesis and emulsifying performance evaluation
Source: Front Nutr. 2024 Jul 9;11:1408937. doi: 10.3389/fnut.2024.1408937 (PMC11265155; doi:10.3389/fnut.2024.1408937)

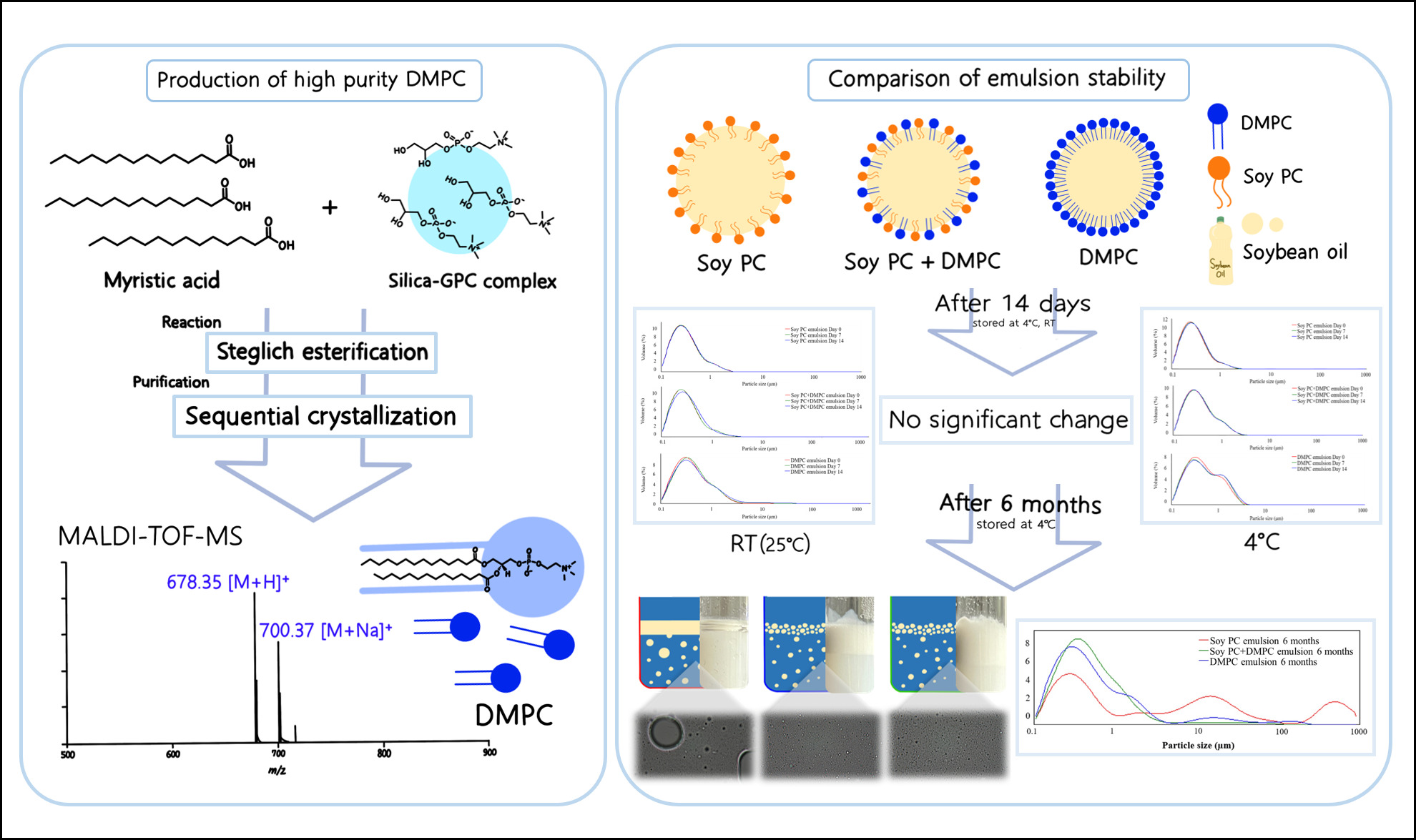

Supplement: Supplementary file 1 [file Image_1.JPEG]
